# Association Between Motor and Cognitive Performances in Elderly With Atrial Fibrillation: Strat-AF Study

**DOI:** 10.3389/fneur.2020.571978

**Published:** 2020-11-13

**Authors:** Emilia Salvadori, Francesco Galmozzi, Francesca Uda, Carmen Barbato, Eleonora Camilleri, Francesca Cesari, Stefano Chiti, Stefano Diciotti, Samira Donnini, Benedetta Formelli, Silvia Galora, Betti Giusti, Anna Maria Gori, Chiara Marzi, Anna Melone, Damiano Mistri, Francesca Pescini, Giovanni Pracucci, Valentina Rinnoci, Cristina Sarti, Enrico Fainardi, Rossella Marcucci, Anna Poggesi

**Affiliations:** ^1^IRCCS Fondazione Don Carlo Gnocchi, Florence, Italy; ^2^Neuroscience Section, NEUROFARBA Department, University of Florence, Florence, Italy; ^3^Department of Experimental and Clinical Medicine, University of Florence, Florence, Italy; ^4^Atherothrombotic Diseases Center, Careggi University Hospital, Florence, Italy; ^5^Central Laboratory, Careggi University Hospital, Florence, Italy; ^6^Department Health Professions, U.O. Research and Development, Careggi University Hospital, Florence, Italy; ^7^Department of Electrical, Electronic, and Information Engineering ‘Guglielmo Marconi’, University of Bologna, Bologna, Italy; ^8^Stroke Unit, Careggi University Hospital, Florence, Italy; ^9^Neuroradiology Unit, Department of Experimental and Clinical Biomedical Sciences, Careggi University Hospital, University of Florence, Florence, Italy

**Keywords:** elderly, atrial fibrillation, cognition, motor performance, gait speed

## Abstract

**Background/Objective:** Growing evidence suggests a close relationship between motor and cognitive abilities, but possible common underlying mechanisms are not well-established. Atrial fibrillation (AF) is associated with reduced physical performance and increased risk of cognitive decline. The study aimed to assess in a cohort of elderly AF patients: (1) the association between motor and cognitive performances, and (2) the influence and potential mediating role of cerebral lesions burden.

**Design:** Strat-AF is a prospective, observational study investigating biological markers for cerebral bleeding risk stratification in AF patients on oral anticoagulants. Baseline cross-sectional data are presented here.

**Setting:** Thrombosis outpatient clinic (Careggi University Hospital).

**Participants:** One-hundred and seventy patients (mean age 77.7 ± 6.8; females 35%).

**Measurements:** Baseline protocol included: neuropsychological battery, motor assessment [Short Physical Performance Battery (SPPB), and walking speed], and brain magnetic resonance imaging (MRI) used for the visual assessment of white matter hyperintensities, lacunar and non-lacunar infarcts, cerebral microbleeds, and global cortical and medial temporal atrophies.

**Results:** Mean Montreal Cognitive Assessment (MoCA) total score was 21.9 ± 3.9, SPPB total score 9.5 ± 2.2, and walking speed 0.9 ± 0.2. In univariate analyses, both SPPB and walking speed were significantly associated with MoCA (*r* = 0.359, *r* = 0.372, respectively), visual search (*r* = 0.361, *r* = 0.322), Stroop (*r* = −0.272, *r* = −0.263), short story (*r* = 0.263, *r* = 0.310), and semantic fluency (*r* = 0.311, *r* = 0.360). In multivariate models adjusted for demographics, heart failure, physical activity, and either stroke history (Model 1) or neuroimaging markers (Model 2), both SPPB and walking speed were confirmed significantly associated with MoCA (Model 1: β = 0.256, β = 0.236; Model 2: β = 0.276, β = 0.272, respectively), visual search (Model 1: β = 0.350, β = 0.313; Model 2: β = 0.344, β = 0.307), semantic fluency (Model 1: β = 0.223, β = 0.261), and short story (Model 2: β = 0.245, β = 0.273).

**Conclusions:** In our cohort of elderly AF patients, a direct association between motor and cognitive functions consistently recurred using different evaluation of the performances, without an evident mediating role of cerebral lesions burden.

## Introduction

Gait is a complex task involving the integration of several brain regions, and high-order cognitive functions are currently recognized to play a role in coordinating and controlling mobility (1, 2). Aging is known to be related to both cognitive decline and reduced physical performance. Although these processes may occur separately, a growing body of literature suggests the presence of a close relationship between cognitive and motor dysfunction. Evidence also indicates that walking impairment could precede cognitive decline, and thereby it may represent a marker of cognitive status and contribute to an early identification of subjects at risk of cognitive impairment (3–5). Possible shared pathological mechanisms underlying this relationship are not well-established, and most studies investigate the hypothesis of a neural overlap determined by the possible engagement of shared neural circuits (6–8).

Among the cognitive abilities, executive functions and processing speed are the most commonly associated with gait dysfunction, with evidence coming both from studies on healthy older adults and on neurologic patients (9, 10). Both neurodegenerative and vascular processes are well-known neurobiological determinants of cognitive decline and could to some extent contribute to motor impairment. Since gait disturbances may differ among the various mechanisms related to the cerebral lesion burden, the interplay between cognitive and motor dysfunctions and the determination of different profiles of gait disturbance among the various subtypes of preclinical or clinical dementia may further contribute to early and differential diagnoses (11–14).

Atrial fibrillation (AF) is a common cardiac arrhythmia associated with a high vascular risk factor profile, cardiac comorbidities, frailty, reduced physical performance, and increased risk of stroke and cognitive decline (15, 16). Besides the occurrence of an acute stroke event, mechanisms such hypoperfusion, inflammation, and endothelial dysfunction may play a role in the association between AF and cognitive decline and may also represent possible common denominators of both vascular and degenerative underlying processes. Furthermore, preliminary data are emerging also on the association between AF and reduced mobility in older adults independently of comorbidities and frailty markers (17–19). Only one recent study by Marino and colleagues directly assessed the association between a reduced gait speed and the presence of cognitive impairment in a population of elderly patients with AF (20). Their results confirmed such association independently from several demographic and clinical characteristics, and the authors concluded that physical changes in gait could be related to declines in the cognitive domains that regulate several gait elements as previously documented in literature on neurologic diseases.

The present study aims to assess in a cohort of elderly patients with a diagnosis of AF and ongoing anticoagulant therapy: (1) the association between motor and cognitive performances, and (2) the influence and potential mediating role of cerebral lesions burden.

## Materials and Methods

The Strat-AF study (stratification of cerebral bleeding risk in AF) is an observational, prospective single-center hospital-based study aimed at improving the prediction of bleeding risk in AF patients under treatment with oral anticoagulants (OACs). Inclusion criteria were age ≥65 years, diagnosis of AF, ongoing OAC with vitamin K antagonists, or direct OACs, and no contraindications to MRI. Ethical approval was obtained by the Ethics Committee of Careggi University Hospital, and all participants gave written informed consent for inclusion before enrolment. Consecutive patients referring to Center of Thrombosis outpatient-clinic of the Careggi University Hospital of Florence and fulfilling inclusion criteria were invited to participate in the study. Clinico-radiological follow-up is still in progress. Study design and methodology have been previously described (21). At enrollment, data on demographic characteristics (age, sex, years of education), previous stroke events, and vascular risk factors and comorbidities (hypertension, diabetes, dyslipidemia, physical activity, smoking habits, alcohol consumption, peripheral arterial disease, ischemic heart disease, myocardial infarction, heart failure) were collected. Furthermore, according to the study protocol, at baseline evaluation all participants underwent a comprehensive cognitive and motor assessment and brain MRI.

### Cognitive and Motor Assessment

The neuropsychological battery included the Montreal cognitive assessment test (MoCA) as a global cognitive functioning test and 6-s level tests for the evaluation of the following domains: verbal memory (Rey Auditory-Verbal Learning Test, short-story test), attention and executive functions (visual search test, Color Word Stroop), and language (semantic verbal fluency test, sentence construction test) (21). Neuropsychological tests' raw scores were corrected for demographics according to the Italian population normative data, and then used in the statistical analyses as continuous variables.

Motor performance assessment included an objective evaluation by means of the Short Physical Performance Battery (SPPB) and walking speed (22). The SPPB is a composite measure designed to assess three aspects of physical performance: standing balance (the ability to stand for up to 10 s with feet in side-by-side, semi-tandem, and tandem positions), gait (time to complete a 4-m walk), and sit-to-stand time (time to rise from a chair five times). Each task is scored out of four, with the scores from the three tests summed to give a total, with a maximum of 12 (best performance) and a minimum of 0 (worst performance).

Walking speed was measured on a flat surface, and patients were instructed to walk for 1 min at their usual speed. Walking speed (meters/seconds) for each patient was calculated.

### Neuroimaging Assessment

Brain MRI have been performed on a 1.5 T MRI (Ingenia, Philips Healthcare, Best, The Netherlands). The MRI protocol included the following sequences: sagittal T1-weighted spin-echo [repetition time (TR) = 547 ms; echo time (TE) = 12 ms; slice thickness = 5 mm; interslice spacing = 0.5 mm; matrix size = 320 × 250; field of view (FOV) = 23 cm × 23 cm; number of signals averaged (NSA) = 1], coronal T2-weighted fast spin-echo (TR = 3347 ms; TE = 110 ms; slice thickness = 5 mm; interslice spacing = 0.5 mm; matrix size = 512 × 322; FOV = 22 cm × 22 cm; NSA = 2); axial fluid-attenuated inversion recovery (FLAIR) [TR = 11,000 ms; TE = 125 ms; inversion time (TI) = 2800 ms; slice thickness = 5 mm; interslice spacing = 0.5 mm; matrix size = 384 × 204; FOV = 23 cm × 23 cm; NSA = 2]; axial gradient-echo T2^*^ (GRE) [TR = 534 ms; TE = 23 ms; flip angle (FA) = 18; slice thickness = 5 mm; interslice spacing = 0.5 mm; matrix size = 256 × 185; FOV = 23 cm × 23 cm; NSA = 1]; axial diffusion weighted imaging (DWI) (TR = 3891 ms; TE = 75 ms; slice thickness = 5 mm; interslice spacing = 0.5 mm; matrix size = 164 × 162; FOV = 23 cm × 23 cm; NSA = 2); gradient-echo 3D T1-weighted (TR = 7.5 ms; TE = 3.4 ms; TI = 950, slice thickness = 1 mm; matrix size = 256 × 241; FOV = 25.6 cm × 25.6 cm; NSA = 1) followed by multiplanar reconstruction (MPR) in axial, coronal, and sagittal planes. Cerebral lesion burden was visually assessed by a trained and experienced rater using validated scales.

Cerebrovascular lesion burden encompassed markers of small vessel disease (SVD) and large vessel disease.

- Non-lacunar infarcts were numerically rated on T1-weighted and FLAIR sequences.

SVD markers were selected and evaluated according to the STRIVE criteria (23), and included:

- White matter hyperintensities (WMH): rated on axial FLAIR sequences using the modified Fazekas scale (24), which defines three different grades of deep WMH severity: mild (single lesions <10 mm; areas of “grouped” lesions <20 mm in any diameter), moderate (single hyperintense lesions between 10 and 20 mm; areas of “grouped” lesions ≥20 mm in any diameter; no more than “connecting bridges” between individual lesions), and severe (single lesions or confluent areas of hyperintensity ≥20 mm in any diameter).

- Cerebral microbleeds (CMB): rated on axial gradient-echo T2^*^ sequences according to the Microbleeds Anatomical Rating Scale (MARS) (25).

- Lacunar infarcts: numerically rated on T2 FLAIR sequences.

Further evaluation of cerebral lesion load, included:

- Global cortical atrophy (GCA): rated on axial T1 or FLAIR sequences using the 0–3 points Pasquier scale (26).

- Medial temporal atrophy (MTA): graded on coronal T1 or FLAIR sequences using the 0–4 points Scheltens visual scale (27).

### Statistical Analyses

Descriptive analyses (frequencies and percentages or means and standard deviations) were carried out to describe the total cohort in terms of demographics, vascular risk factors, comorbidities, history of stroke, and motor, cognitive, and neuroimaging characteristics. For analyses purposes, neuroimaging characteristics were dichotomized as follows: non-lacunar infarcts absent *vs*. ≥1; WMH absent-mild (Fazekas score 0–1) vs. moderate-severe (Fazekas score 2–3); cerebral microbleeds absent vs. ≥1; lacunar infarcts absent vs. ≥1; global cortical atrophy absent-mild (Pasquier score 0–1) vs. moderate-severe (Pasquier score 2–3); and mean left and right MTA Scheltens score 0–1 vs. 2–4.

Univariate analyses (Pearson's *r* or point-biserial *r*_pb_ correlations) were employed to evaluate the association between the performances in cognitive and motor tests, and demographics (age, sex, and education), vascular risk factors or comorbidities (hypertension, diabetes, dyslipidemia, physical activity, smoking habits, alcohol consumption, peripheral arterial disease, ischemic heart disease, myocardial infarction, heart failure), history of stroke, and neuroimaging characteristics. Results on the effects of demographics and vascular risk factors or comorbidities will be further used to decide for their introduction within multivariate models of analyses as potential confounders.

For all univariate analyses a conservative significance threshold of 0.01 was applied.

Independent multivariate linear regression models were used to evaluate the association between each cognitive test, considered as the dependent variable, and SPPB total score or walking speed as the main independent variable controlling for the influence of the cerebral lesions burden considered either as clinical history of stroke in Model 1 or as MRI markers (WMH, CMB, lacunar and non-lacunar infarcts, GCA, and MTA) in Model 2. All multivariate linear regressions used a full model, without any selection procedure for the included independent variables, and Bonferroni corrections for multiple comparisons were applied at the significance threshold of 0.05.

All analyses were done using the SPSS software version 25.

## Results

From September 2017 to March 2019, out of the 194 subjects enrolled in the Strat-AF study, 170 (mean age 77.7 ± 6.8 years, 35% females) completed the baseline MRI protocol and were included in the present study. Demographic, vascular risk factors, comorbidities, motor, cognitive, and neuroimaging characteristics are shown in Table 1. Thirty-eight (22%) patients had history of stroke, the mean SPPB total score was 9.5 ± 2.19, mean walking speed 0.9 ± 0.21, and the mean MoCA score was 21.9 ± 3.9. Concerning the distributions of neuroimaging characteristics: a moderate to severe degree of WMH was present in the 21% of the total cohort, lacunar infarct in 32%, and CMB in 28%. A moderate to severe degree of cortical atrophy was observed in 78% of patients. Non-lacunar infarcts in 31%, and a mean MTA score ≥ 2 in 64%.

**Table 1:** Demographic, history of stroke, vascular risk factors, comorbidities, motor, cognitive, and neuroimaging characteristics of the Strat-AF study cohort (means and standard deviations or frequencies and percentages).

		**Total cohort**
		***n* = 170**
Age (years)		77.7 ± 6.8
Sex (female)		60 (35%)
Education (years)		9.4 ± 4.3
History of stroke		38 (22%)
Hypertension		140 (82%)
Diabetes		22 (13%)
Dyslipidemia		87 (51%)
Physical activity		60 (35%)
Smoking habits		105 (62%)
Alcohol consumption		91 (53%)
Peripheral arterial disease		14 (8%)
Ischemic heart disease		18 (11%)
Myocardial infarction		15 (9%)
Heart failure		25 (15%)
CHA_2_DS_2_-VASc score		3.7 ± 1.5
Type of oral anticoagulant therapy	Vitamin K antagonists Direct oral anticoagulants	52 (31%) 118 (69%)
SPPB total score		9.5 ± 2.2
Walking speed		0.9 ± 0.2
MoCA		21.9 ± 3.9
Visual search		36.3 ± 7.4
Stroop (time to complete)		28.2 ± 28.7
RAVLT (immediate recall)		36.7 ± 8.5
RAVLT (delayed recall)		6.7 ± 2.6
Short story		14.2 ± 4.7
Sentence construction		19.5 ± 6.4
Semantic verbal fluency		42.4 ± 9.3
White matter hyperintensities	Moderate-severe	35 (21%)
Non-lacunar infarcts	≥1	53 (31%)
Lacunar infarcts	≥1	54 (32%)
Cerebral microbleeds	≥1	48 (28%)
Cortical atrophy	Moderate-severe	133 (78%)
Medial temporal atrophy	Mean ≥ 2	108 (64%)

*SPPB, short physical performance battery; MoCA, montreal cognitive assessment, RAVLT, Rey auditory-verbal learning test*.

Concerning the influence of demographics on cognitive and motor performances, older age was significantly associated with a worse performance in both SPPB total score (*r* = −0.309 *p* = 0.001) and walking speed (*r* = −0.409 *p* = 0.001), and a lesser level of education was significantly associated with worse performances in MoCA (*r* = 0.200 *p* = 0.009) and walking speed (*r* = 0.215 *p* = 0.005). Compared to males, females had a statistically significant worse performance in MoCA (*r*_pb_ = −0.338 *p* = 0.001), short story (*r*_pb_ = −0.329 *p* = 0.001), and semantic verbal fluency (*r*_pb_ = −0.252 *p* = 0.001), as well as in both motor indexes (*r*_pb_ = −0.245 *p* = 0.001 for SPPB, *r*_pb_ = −0.345 *p* = 0.001 for walking speed). All demographic variables were then introduced as independent variables in multivariate models of analysis.

Taking into account the effect of vascular risk factors and comorbidities on cognitive and motor performances, univariate analyses showed a significant association between heart failure and SPPB total score (*r*_pb_ = −0.201 *p* = 0.009), and between physical activity and performances in MoCA (*r*_pb_ = 0.231 *p* = 0.003), SPPB (*r*_pb_ = 0.278 *p* = 0.001), and walking speed (*r*_pb_ = 0.349 *p* = 0.001). Heart failure and physical activity were then introduced as independent variables in multivariate models of analysis.

Table 2 shows the univariate associations between motor and cognitive performances. Both indexes of motor performance were significantly associated with MoCA, visual search, Stroop, short story, and semantic verbal fluency scores.

**Table 2:** Association between cognitive and motor performances (univariate analyses: Pearson's *r*).

**Cognitive domains**	**Cognitive measures**	**Short physical performance battery total score**	**Walking speed**
Global cognitive functioning	Montreal cognitive assessment	0.359^*^	0.372^*^
Attention and executive functions	Visual search	0.361^*^	0.322^*^
	Stroop (time)	−0.272^*^	−0.263^*^
Memory	Rey Auditory-Verbal Learning Test (immediate recall)	0.172	0.132
	Rey Auditory-Verbal Learning Test (delayed recall)	0.038	0.063
	Short story	0.263^*^	0.310^*^
Language	Sentence construction	0.146	0.166
	Semantic verbal fluency	0.311^*^	0.360^*^

* *Statistically significant at p < 0.01. Bold values are statistically significant*.

The influence of history of stroke on cognitive and motor performances, and its mediating role on their association, was further evaluated. In Table 3, the presence of history of stroke resulted significantly associated with a worse performance on MoCA, Stroop, and sentence construction tests, as well as with a reduced walking speed. Multivariate linear regression models on the association between cognitive tests and indexes of motor performance taking into account the effect of stroke history are shown in Table 4 (Models 1). Even after controlling for history of stroke, both the motor indexes were significantly associated with MoCA, visual search, and semantic fluency scores in all adjusted models. The presence of stroke history was confirmed to be another statistically significant predictor of MoCA performance in both Models 1, while its associations with Stroop (β = 0.183, *p* = 0.017 for SPPB, β = 0.183, *p* = 0.020 for walking speed) and sentence construction (β = −0.184, *p* = 0.020 for SPPB, β = −0.176, *p* = 0.028 for walking speed) lost significance from a statistical point of view according to the correction for multiple comparisons.

**Table 3:** Cognitive and motor performances and their associations with history of stroke and neuroimaging characteristics (univariate analyses: Pearson's *r* and point-biserial *r*_pb_ correlations).

		**History of stroke**	**WMH**	**Non-lacunar infarcts**	**Lacunar infarcts**	**CMB**	**GCA**	**MTA**
		**Present**	**Moderate-severe**	**≥1**	**≥1**	**≥1**	**Moderate-severe**	**Mean ≥ 2**
Cognitive measures	MoCA	–0.334^*^	0.067	−0.231^*^	0.002	−0.046	0.052	0.027
	Visual search	–0.078	0.043	−0.065	0.032	−0.239^*^	−0.127	−0.022
	Stroop (time)	0.222^*^	−0.031	0.104	−0.057	0.024	0.025	0.091
	RAVLT (immediate recall)	–0.185	−0.019	−0.135	−0.015	−0.156	−0.028	0.004
	RAVLT (delayed recall)	–0.141	0.023	−0.046	0.086	0.048	0.096	0.023
	Short story	–0.147	0.157	−0.149	0.036	0.048	0.007	0.063
	Sentence construction	–0.208^*^	−0.013	−0.104	−0.057	0.044	−0.004	−0.143
	Semantic verbal fluency	–0.197	0.059	−0.190	0.064	−0.073	−0.007	−0.042
Motor indexes	SPPB total score	−0.177	−0.027	−0.141	0.024	−0.084	−0.117	−0.157
	Walking speed	−0.236^*^	−0.119	−0.192	−0.040	−0.033	−0.159	−0.143

* *Statistically significant at p < 0.01*.

*SPPB, short physical performance battery; MoCA, montreal cognitive assessment, RAVLT, Rey auditory-verbal learning test; WMH, white matter hyperintensities, CMB, cerebral microbleeds, GCA, global cortical atrophy; MTA, medial temporal atrophy. Bold values are statistically significant*.

**Table 4:** Association between the performances in cognitive tests and motor indexes adjusted for history of stroke (Model 1) or neuroimaging characteristics (Model 2), and demographics, heart failure, and physical activity (all models).

**Cognitive measure (dependent)**	**Motor index (independent)**	**Model 1**^**#**^		**Model 2**^**##**^	
		**B standard**	**Other statistically significant independent variables**	**B standard**	**Other statistically significant independent variables**
MoCA	SPPB total score	0.256^*^ **–**0.206^*^ **–**0.262^*^	Sex History of stroke	0.276^**^	
	Walking speed	0.236^*^ **–**0.257^*^	History of stroke	0.272^**^	
Visual search	SPPB total score	0.350^*^		0.344^**^ −0.226^**^	Cerebral microbleeds
	Walking speed	0.313^*^		0.307^**^ –0.245^**^	Cerebral microbleeds
Stroop (time to complete)	SPPB total score	−0.196		−0.212	
	Walking speed	−0.152		−0.199	
RAVLT (immediate recall)	SPPB total score	0.187		0.189	
	Walking speed	0.134		0.119	
RAVLT (delayed recall)	SPPB total score	0.034		0.051	
	Walking speed	0.057		0.077	
Short story	SPPB total score	0.197 **–**0.248^*^	Sex	0.245^**^	
	Walking speed	0.225		0.273^**^	
Sentence construction	SPPB total score	0.100		0.094	
	Walking speed	0.107		0.112	
Semantic verbal fluency	SPPB total score	0.223^*^		0.235	
	Walking speed	0.261^*^		0.273^**^	

# *Multivariate independent models of linear regression adjusted for demographics (age, education, sex), heart failure, physical activity and history of stroke*.

## *Multivariate independent models of linear regression adjusted for demographics (age, education, sex), heart failure, physical activity, and neuroimaging characteristics (white matter hyperintensities; non-lacunar infarcts; lacunar infarcts; cerebral microbleeds; global cortical atrophy; medial temporal atrophy)*.

* *Statistically significant at p < 0.007 (Bonferroni correction for multiple comparisons)*.

** *Statistically significant at p < 0.004 (Bonferroni correction for multiple comparisons)*.

*MoCA, montreal cognitive assessment, RAVLT, Rey auditory-verbal learning test, SPPB, short physical performance battery. Bold and italics values represent the statistically significant beta coefficients of the corresponding variables of the “Other statistically significant independent variables” column*.

Table 3 shows the association between neuroimaging characteristics and measures of cognitive and motor performances by means of univariate correlational analyses. Among the neuroimaging characteristics, the presence of non-lacunar infarcts was significantly associated with MoCA scores and the presence of CMB with visual search test scores. In both cases, the statistical direction of the association indicated a worse cognitive performance in patients having at least one cerebral lesion.

Results from the independent multivariate linear regression models on the association between each cognitive test and indexes of motor performance controlling for the effect of neuroimaging characteristics are shown in Table 4 (Models 2). The associations of both motor indexes with MoCA, and visual search, and of walking speed with semantic fluency, were further confirmed to be statistically significant independently on the effect of neuroimaging characteristics. Furthermore, both motor indexes were significantly associated with short story. In multivariate models, the presence of CMB was confirmed to be another statistically significant predictor of visual search test performance in both Models 2, while the association between non-lacunar infarcts and MoCA remained as a trend but lost statistical significance according to the correction for multiple comparisons (β = −0.160, *p* = 0.030 for SPPB, β = −0.156, *p* = 0.038 for walking speed).

Finally, taking into account the influence of the other independent variables in multivariate models, sex was confirmed as another statistically significant predictor of MoCA and short-story tests only in Models 1 among demographics, while heart failure and physical activity were not statistically significant (Table 4).

## Discussion

In this sample of hospital-based older adults with diagnosis of AF and ongoing anticoagulant therapy for the primary or secondary prevention of thromboembolism, we examined the relationship between mobility and cognition by the use of several objective measures of motor ability and high order cognitive functions. Our results showed a consistent association between motor and cognitive performances in our cohort. Specifically, motor abilities, herein evaluated both as a composite measure of physical performance and a gait speed measure, resulted as being associated with global cognitive functioning, attention, prose memory, and verbal fluency.

This evidence is in line with data from recent systematic reviews and meta-analyses of cross-sectional studies that support an association between mobility measures and cognitive assessments in healthy older adults (1, 7). Convergent evidence suggests that individuals with better mobility, mainly evaluated by means of gait speed, perform better on assessments of global cognition, executive function, processing speed, memory, and language, thus reinforcing the hypothesis of a multifaceted influence of high-order cognitive abilities.

Among cognitive domains, attention and executive functions have received considerable interest in studies on the association between mobility and cognition (9, 10). The rationale of this interest relies on several potential factors, for example, the influence of psychomotor and processing speed on mobility, the attentional and executive demands associated with motor regulation, and the subcortical brain networks involvement in frontal lobes functions. Our results seemed to confirm a reliable association between mobility and cognitive performances related to attention and executive processes. Among the cognitive tests taken into consideration in the present study, those with the most consistent association with both mobility indexes were MoCA and visual search. MoCA is a screening test suggested by the National Institute for Neurological Disorders and Stroke and the Canadian Stroke Network (NINDS-CSN) to harmonize standards for the evaluation of vascular cognitive impairment, because it includes several items assessing executive functions, attention and concentration (28). The visual search test is a simple timed selective digit cancellation task that involves focused and sustained attention together with the attentional control of the speed/accuracy trade-off.

Considering the specificity of our cohort, that is, elderly AF patients, our results were in line with those of the only study that showed a direct association between gait and cognition in this population (20). Marino and colleagues found that, among the 1,185 AF participants aged ≥65 years, those with low gait speed were significantly more likely to have cognitive impairment independently from several demographic and clinical characteristics. Also in our cohort, the association between motor and cognitive performances was confirmed to be independent of demographics, vascular risk factors, and comorbidities.

Furthermore, we introduced the potential effect of cerebral lesions burden within this association, and found its persistence independently of these factors.

One recent study by Conen and et al. (29) evaluated the relationships between cognitive function, assessed by MoCA, and vascular brain lesions in a large sample of patients with AF (*N* = 1,737, mean age 73 ± 8 years). Their results showed a high burden of vascular brain lesions, mostly related to clinically silent infarcts. In our cohort, we found a comparable vascular lesion burden, taking into account also that actually our sample is 4 years older on average. Taking into account the impact of all different lesions types on cognition, Conen and colleagues found that only large infarcts were significantly associated with a lower MoCA performance. In line with this, also in our cohort we found that non-lacunar infarcts, or history of stroke, influenced MoCA performance.

Interestingly, in our cohort CMB seemed to consistently influence the performance on the visual search test. CMBs are recognized as MRI markers of SVD, and an increasing number of studies have linked their presence with cognitive decline (30, 31). Evidence suggest that CMB may play a role in vascular cognitive impairment, with preliminary data of an influence on tests sensitive to executive dysfunction, consistent with a possible effect of CMB on frontal-subcortical circuits. Results in our cohort seemed consistent with an influence of CMB on focused and sustained attention. Also, CMB prevalence within our sample seemed in line with rates reported in studies on patients with vascular cognitive impairment, and was slightly higher than the prevalence of 21% reported for the 1,490 adult AF participants of the CROMIS-2 study (31–34).

Limitations of our study need to be considered. The present study is based on a secondary analysis of the Strat-AF study, which is a single center study. The sample size imposes caution in the interpretation of our results that cannot be generalized to other populations. Moreover, the cross-sectional design of the analysis limits further interpretations of the direction of the association, precluding causal inferences regarding the relationship between gait and cognition. Longitudinal data collection is ongoing, and further prospective analysis will be conducted to elucidate the role of motor performance as potential predictor of cognitive impairment.

Another limitation is the neuroimaging assessment based on visual evaluations of brain MRI. Compared to the use of quantitative brain volumetric and morphometric measures, our approach might have reduced accuracy in the quantification of cerebral lesions burden. Also, CMB have been evaluated on traditional gradient recalled-echo (GRE) MRI sequences. The inclusion in the protocol of sequences with higher resolution, that is, the susceptibility-weighted imaging (SWI), would have increased the sensitivity in detecting CMB and probably would have reinforced the association with cognitive and motor performances. Finally, related to our limited sample size, we have used a dichotomic approach for the evaluation of cerebral lesions burden and this might have potentially underestimated their effect on clinical outcomes.

Recognized markers for neurodegeneration, specifically Alzheimer Disease, such as cerebrospinal fluid biomarkers (amyloid-β and tau proteins concentrations) and positron emission tomography imaging (FDG and amyloid) are not available in our cohort (35). The only marker for neurodegeneration was MTA, and this was not associated with motor and cognitive performances. Of note, nearly two-thirds of our sample scored 2 or more on MTA. Despite the link between AF and cerebral volume loss remains unclear, possible hypotheses include mechanisms such as hypoperfusion, inflammation, and endothelial dysfunction, and thus a possible close interplay between vascular and neurodegenerative processes.

Last but not least, our approach to gait evaluation was based on qualitative measures, which on one side are feasible in clinical setting with minimal costs and time, on the other might be less sensitive than quantitative gait analysis (e.g., pace, rhythm, variability, asymmetry, and postural control).

In conclusion, our study has confirmed the existence of a direct and strong association between motor and cognitive performances in a population of elderly AF patients, also taking into account the effect and potential mediating role of cerebral lesion burden. The determinants and common mechanisms of this relationship remain unexplained, and further studies are needed to better characterize the association.

## Data Availability Statement

The datasets presented in this article are not readily available because Data are not freely available. Requests to access the datasets should be directed to Anna Poggesi, anna.poggesi@unifi.it.

## Ethics Statement

The studies involving human participants were reviewed and approved by Comitato Etico Area Vasta Centro—Careggi University Hospital. The patients/participants provided their written informed consent to participate in this study.

## Author Contributions

AP, ES, and FG: study concept and design. FG, FU, CB, EC, SC, SDo, BF, SG, AM, DM, and VR: acquisition of data. AP, ES, FG, and FU: analysis and interpretation of data. AP, ES, and FG: preparation of the manuscript. EF, FC, SDi, BG, AG, CM, FP, GP, CS, and RM: critical revision of the manuscript. All authors contributed to the article and approved the submitted version.

## Conflict of Interest

The authors declare that the research was conducted in the absence of any commercial or financial relationships that could be construed as a potential conflict of interest.
